# Bis{2-[(2-pyrid­yl)imino­meth­yl]phenolato}copper(II)

**DOI:** 10.1107/S1600536809026051

**Published:** 2009-07-11

**Authors:** Jinling Miao, Zhitong Zhao, Hongwei Chen, Daqi Wang, Yong Nie

**Affiliations:** a School of Chemistry and Chemical Engineering, University of Jinan, Jinan 250022, People’s Republic of China; b College of Chemistry and Chemical Engineering, Liaocheng University, Liaocheng 252059, People’s Republic of China.

## Abstract

In the title compound, [Cu(C_12_H_9_N_2_O)_2_], the Cu^II^ atom lies on a crystallographic inversion center and has a nearly square-planar geometry. The Cu^II^ center coordinates to the phenolic O and azomethine N atoms of the two symmetry-related 2-[(2-pyrid­yl)imino­meth­yl]phenolate ligands. The pyridyl N atoms do not coordinate to the Cu^II^ atom but participate in intra­molecular C—H⋯N hydrogen bonding. π–π stacking between the benzene rings and between the pyridyl rings [centroid–centroid distances 3.8142 (5) and 3.8142 (5) Å, respectively] links the mol­ecules into a chain propagating parallel to [100].

## Related literature

For the preparation of the title compound by an electrochemical method, see: Castineiras *et al.* (1989 ▶), and by a solution method, see: Parashar *et al.* (1988 ▶). For the crystal structures of related compounds, see: Castineiras *et al.* (1989 ▶).
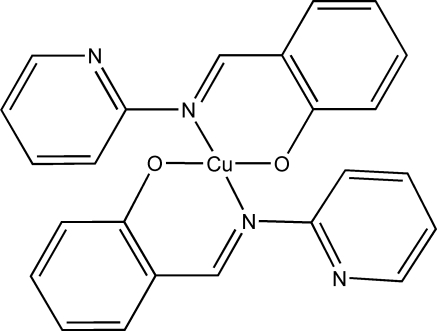


## Experimental

### 

#### Crystal data

[Cu(C_12_H_9_N_2_O)_2_]
*M* *_r_* = 457.96
Triclinic, 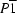

*a* = 3.8142 (5) Å
*b* = 11.217 (1) Å
*c* = 11.9001 (12) Å
α = 106.884 (2)°
β = 90.374 (1)°
γ = 90.289 (1)°
*V* = 487.16 (9) Å^3^
*Z* = 1
Mo *K*α radiation
μ = 1.15 mm^−1^
*T* = 298 K
0.41 × 0.17 × 0.15 mm

#### Data collection

Bruker SMART 1000 CCD area-detector diffractometer
Absorption correction: multi-scan (*SADABS*; Bruker, 2001 ▶) *T*_min_ = 0.650, *T*_max_ = 0.846
2547 measured reflections
1695 independent reflections
1481 reflections with *I* > 2σ(*I*)
*R*_int_ = 0.015

#### Refinement

*R*[*F* ^2^ > 2σ(*F* ^2^)] = 0.033
*wR*(*F* ^2^) = 0.077
*S* = 1.07
1695 reflections
142 parameters
H-atom parameters constrained
Δρ_max_ = 0.27 e Å^−3^
Δρ_min_ = −0.29 e Å^−3^

### 

Data collection: *SMART* (Bruker, 2001 ▶); cell refinement: *SAINT* (Bruker, 2001 ▶); data reduction: *SAINT*; program(s) used to solve structure: *SHELXS97* (Sheldrick, 2008 ▶); program(s) used to refine structure: *SHELXL97* (Sheldrick, 2008 ▶); molecular graphics: *SHELXTL* (Sheldrick, 2008 ▶); software used to prepare material for publication: *SHELXTL*.

## Supplementary Material

Crystal structure: contains datablocks I, global. DOI: 10.1107/S1600536809026051/pv2176sup1.cif

Structure factors: contains datablocks I. DOI: 10.1107/S1600536809026051/pv2176Isup2.hkl

Additional supplementary materials:  crystallographic information; 3D view; checkCIF report

## Figures and Tables

**Table 1:** Hydrogen-bond geometry (Å, °)

*D*—H⋯*A*	*D*—H	H⋯*A*	*D*⋯*A*	*D*—H⋯*A*
C1—H1⋯N1	0.93	2.29	2.684 (3)	105

**Fig. 1.**
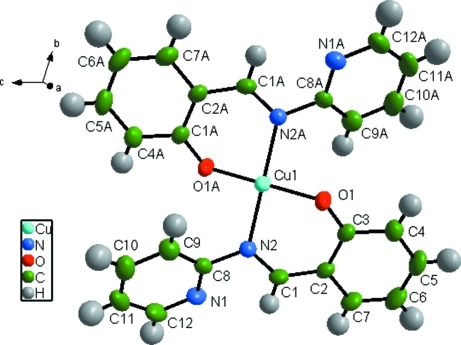
The molecular structure, with atom labels and 25% probability thermal ellipsoids.

**Fig. 2.**
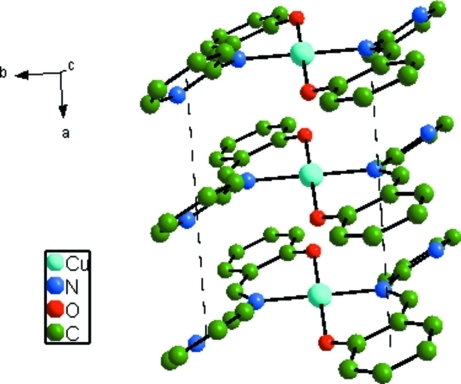
The one-dimensional chain constructed by the π-π stacking.

## supplementary crystallographic information

### Comment

The Schiff base, *N*-salicylidene 2-aminopyridine, has been widely studied as a potential tridentate ligand. The title compound has been prepared using an electrochemical method by Castineiras *et al.* (1989) starting from *N*-salicylidene 2-aminopyridine and copper. Parashar *et al.* (1988) reported that refluxing a mixture of Cu(OAc)_2_ (OAc = acetato) and *N*-salicylidene 2-aminopyridine in a 1:2 molar ratio resulted in a green complex having the same formula but with an octahedral geometry deduced from spectroscopic properties. We have found that a simple method of solution diffusion produces the brown title compound.

As shown in Fig. 1, the copper atom lies on a crystallographic inversion center and has a square planar geometry. The copper center coordinates to the phenolic oxygen and the azomethine nitrogen atoms of the two symmetry related groups. The pyridyl nitrogen atoms do not coordinate to the copper. The Cu—O bond lengths are 1.9212 (17) Å, and the Cu—N bond lengths are 2.0216 (19) Å, respectively, all similar to those reported in the related structures (Castineiras *et al.*, 1989).

The interplane dihedral angles are found to be as follows: 31.60 (7)° between the N_2_O_2_ plane and the benzene ring, 54.28 (7)° between the N_2_O_2_ plane and the pyridyl ring, and 22.75 (9)° between the benzene and the pyridyl ring. The intramolecular hydrogen bond C1—H1···N1 (2.684 (3) Å, 105°, Table 1) further stabilizes the whole structure. The *π*-*π* stacking between the benzene rings (centroid to centroid, 3.8142 (5) Å) and the pyridyl rings (centroid to centroid, 3.8142 (5) Å) links the molecules into a one-dimensional chain (Fig. 2).

### Experimental

To a green solution of salicylaldehyde (23 mg, 0.19 mmol) and Cu(OAc)_2_.H_2_O (11 mg, 0.05 mmol) in ethanol (7 ml) was added slowly a solution of 2-aminopyridine (21 mg, 0.22 mmol) in ethanol (1 ml). The resulting mixture was allowed to stand still and brown crystalline needles were grown after 1 day. IR (KBr): *v* = 3435, 1611, 1444, 1326, 1187 cm ^-1^.

### Refinement

All H-atoms were positioned geometrically and refined using a riding model with d(C—H) = 0.93 Å, *U*_iso_=1.2*U*_eq_ (C).

### Crystal data

**Table:**

[Cu(C_12_H_9_N_2_O)_2_]	*Z* = 1
*M**_r_* = 457.96	*F*(000) = 235
Triclinic, *P*1	*D*_x_ = 1.561 Mg m^−^^3^
Hall symbol: -P 1	Mo *K*α radiation, λ = 0.71073 Å
*a* = 3.8142 (5) Å	Cell parameters from 1475 reflections
*b* = 11.217 (1) Å	θ = 2.2–27.5°
*c* = 11.9001 (12) Å	µ = 1.15 mm^−^^1^
α = 106.884 (2)°	*T* = 298 K
β = 90.374 (1)°	Needle, brown
γ = 90.289 (1)°	0.41 × 0.17 × 0.15 mm
*V* = 487.16 (9) Å^3^	

### Data collection

**Table:**

Bruker SMART 1000 CCD area-detector diffractometer	1695 independent reflections
Radiation source: fine-focus sealed tube	1481 reflections with *I* > 2σ(*I*)
graphite	*R*_int_ = 0.015
φ and ω scans	θ_max_ = 25.0°, θ_min_ = 1.8°
Absorption correction: multi-scan (*SADABS*; Bruker, 2001)	*h* = −4→4
*T*_min_ = 0.650, *T*_max_ = 0.846	*k* = −13→13
2547 measured reflections	*l* = −9→14

### Refinement

**Table:**

Refinement on *F*^2^	Primary atom site location: structure-invariant direct methods
Least-squares matrix: full	Secondary atom site location: difference Fourier map
*R*[*F*^2^ > 2σ(*F*^2^)] = 0.033	Hydrogen site location: inferred from neighbouring sites
*wR*(*F*^2^) = 0.077	H-atom parameters constrained
*S* = 1.07	*w* = 1/[σ^2^(*F*_o_^2^) + (0.0266*P*)^2^ + 0.355*P*] where *P* = (*F*_o_^2^ + 2*F*_c_^2^)/3
1695 reflections	(Δ/σ)_max_ < 0.001
142 parameters	Δρ_max_ = 0.27 e Å^−^^3^
0 restraints	Δρ_min_ = −0.29 e Å^−^^3^

### Fractional atomic coordinates and isotropic or equivalent isotropic displacement parameters (Å^2^)

**Table:**

	*x*	*y*	*z*	*U*_iso_*/*U*_eq_	
Cu1	0.5000	0.5000	0.5000	0.03882 (17)	
N1	0.2225 (6)	0.15806 (19)	0.55001 (19)	0.0400 (5)	
N2	0.4891 (5)	0.31537 (17)	0.48298 (17)	0.0305 (5)	
O1	0.8250 (5)	0.46999 (15)	0.37229 (15)	0.0413 (5)	
C1	0.5395 (7)	0.2344 (2)	0.3821 (2)	0.0331 (6)	
H1	0.4927	0.1517	0.3774	0.040*	
C2	0.6590 (7)	0.2583 (2)	0.2773 (2)	0.0325 (6)	
C3	0.7979 (7)	0.3757 (2)	0.2770 (2)	0.0326 (6)	
C4	0.9196 (7)	0.3865 (3)	0.1691 (2)	0.0389 (6)	
H4	1.0121	0.4623	0.1658	0.047*	
C5	0.9057 (7)	0.2886 (3)	0.0689 (2)	0.0459 (7)	
H5	0.9870	0.2994	−0.0010	0.055*	
C6	0.7726 (8)	0.1734 (3)	0.0696 (2)	0.0494 (7)	
H6	0.7657	0.1072	0.0011	0.059*	
C7	0.6524 (8)	0.1595 (2)	0.1727 (2)	0.0427 (7)	
H7	0.5635	0.0825	0.1738	0.051*	
C8	0.4001 (6)	0.2651 (2)	0.5769 (2)	0.0316 (6)	
C9	0.5106 (7)	0.3277 (3)	0.6892 (2)	0.0412 (6)	
H9	0.6317	0.4030	0.7045	0.049*	
C10	0.4374 (8)	0.2761 (3)	0.7780 (3)	0.0494 (7)	
H10	0.5085	0.3160	0.8547	0.059*	
C11	0.2574 (8)	0.1647 (3)	0.7520 (3)	0.0509 (8)	
H11	0.2045	0.1278	0.8105	0.061*	
C12	0.1583 (8)	0.1094 (3)	0.6381 (3)	0.0491 (8)	
H12	0.0394	0.0335	0.6208	0.059*	

### Atomic displacement parameters (Å^2^)

**Table:**

	*U*^11^	*U*^22^	*U*^33^	*U*^12^	*U*^13^	*U*^23^
Cu1	0.0584 (3)	0.0241 (2)	0.0318 (3)	−0.0022 (2)	0.0154 (2)	0.00443 (18)
N1	0.0457 (14)	0.0298 (11)	0.0452 (13)	−0.0023 (10)	0.0071 (11)	0.0116 (10)
N2	0.0343 (12)	0.0261 (10)	0.0306 (11)	−0.0019 (9)	0.0037 (9)	0.0073 (9)
O1	0.0597 (13)	0.0304 (9)	0.0301 (10)	−0.0079 (9)	0.0143 (9)	0.0029 (8)
C1	0.0363 (15)	0.0233 (12)	0.0375 (14)	0.0001 (10)	0.0005 (11)	0.0054 (11)
C2	0.0341 (14)	0.0286 (13)	0.0312 (13)	0.0052 (11)	0.0027 (11)	0.0030 (10)
C3	0.0335 (14)	0.0330 (13)	0.0290 (13)	0.0046 (11)	0.0036 (11)	0.0051 (11)
C4	0.0384 (16)	0.0444 (15)	0.0339 (14)	−0.0010 (12)	0.0047 (12)	0.0112 (12)
C5	0.0437 (17)	0.065 (2)	0.0258 (14)	0.0025 (14)	0.0037 (12)	0.0081 (13)
C6	0.0526 (19)	0.0517 (18)	0.0318 (15)	0.0027 (14)	0.0007 (13)	−0.0071 (13)
C7	0.0480 (17)	0.0340 (14)	0.0391 (15)	0.0012 (12)	0.0020 (13)	−0.0006 (12)
C8	0.0324 (14)	0.0280 (12)	0.0363 (14)	0.0036 (10)	0.0045 (11)	0.0120 (11)
C9	0.0401 (16)	0.0438 (16)	0.0404 (16)	−0.0012 (12)	−0.0032 (12)	0.0136 (13)
C10	0.0501 (18)	0.064 (2)	0.0370 (16)	0.0118 (15)	0.0021 (13)	0.0186 (14)
C11	0.0545 (19)	0.0555 (19)	0.0544 (19)	0.0185 (15)	0.0182 (15)	0.0336 (16)
C12	0.0541 (19)	0.0367 (15)	0.063 (2)	0.0027 (13)	0.0179 (15)	0.0240 (14)

### Geometric parameters (Å, °)

**Table:**

Cu1—O1	1.9212 (17)	C4—H4	0.9300
Cu1—O1^i^	1.9212 (17)	C5—C6	1.388 (4)
Cu1—N2^i^	2.0216 (19)	C5—H5	0.9300
Cu1—N2	2.0216 (19)	C6—C7	1.363 (4)
N1—C8	1.330 (3)	C6—H6	0.9300
N1—C12	1.339 (3)	C7—H7	0.9300
N2—C1	1.294 (3)	C8—C9	1.379 (4)
N2—C8	1.433 (3)	C9—C10	1.373 (4)
O1—C3	1.310 (3)	C9—H9	0.9300
C1—C2	1.426 (3)	C10—C11	1.376 (4)
C1—H1	0.9300	C10—H10	0.9300
C2—C7	1.406 (3)	C11—C12	1.366 (4)
C2—C3	1.419 (3)	C11—H11	0.9300
C3—C4	1.406 (3)	C12—H12	0.9300
C4—C5	1.366 (4)		
			
O1—Cu1—O1^i^	180.000 (1)	C4—C5—H5	119.4
O1—Cu1—N2^i^	90.50 (8)	C6—C5—H5	119.4
O1^i^—Cu1—N2^i^	89.50 (7)	C7—C6—C5	118.6 (3)
O1—Cu1—N2	89.50 (7)	C7—C6—H6	120.7
O1^i^—Cu1—N2	90.50 (8)	C5—C6—H6	120.7
N2^i^—Cu1—N2	180.000 (1)	C6—C7—C2	121.8 (3)
C8—N1—C12	116.8 (2)	C6—C7—H7	119.1
C1—N2—C8	115.7 (2)	C2—C7—H7	119.1
C1—N2—Cu1	120.87 (16)	N1—C8—C9	123.6 (2)
C8—N2—Cu1	123.30 (15)	N1—C8—N2	117.7 (2)
C3—O1—Cu1	123.48 (16)	C9—C8—N2	118.7 (2)
N2—C1—C2	127.1 (2)	C10—C9—C8	118.3 (3)
N2—C1—H1	116.4	C10—C9—H9	120.9
C2—C1—H1	116.4	C8—C9—H9	120.9
C7—C2—C3	119.6 (2)	C9—C10—C11	119.2 (3)
C7—C2—C1	118.2 (2)	C9—C10—H10	120.4
C3—C2—C1	122.1 (2)	C11—C10—H10	120.4
O1—C3—C4	120.4 (2)	C12—C11—C10	118.4 (3)
O1—C3—C2	122.7 (2)	C12—C11—H11	120.8
C4—C3—C2	116.9 (2)	C10—C11—H11	120.8
C5—C4—C3	121.8 (3)	N1—C12—C11	123.8 (3)
C5—C4—H4	119.1	N1—C12—H12	118.1
C3—C4—H4	119.1	C11—C12—H12	118.1
C4—C5—C6	121.3 (3)		
			
O1—Cu1—N2—C1	−29.5 (2)	C3—C4—C5—C6	−0.5 (4)
O1^i^—Cu1—N2—C1	150.5 (2)	C4—C5—C6—C7	0.5 (4)
O1—Cu1—N2—C8	155.47 (19)	C5—C6—C7—C2	0.2 (4)
O1^i^—Cu1—N2—C8	−24.53 (19)	C3—C2—C7—C6	−0.8 (4)
N2^i^—Cu1—O1—C3	−138.9 (2)	C1—C2—C7—C6	−177.8 (3)
N2—Cu1—O1—C3	41.1 (2)	C12—N1—C8—C9	−1.5 (4)
C8—N2—C1—C2	−174.2 (2)	C12—N1—C8—N2	176.5 (2)
Cu1—N2—C1—C2	10.4 (4)	C1—N2—C8—N1	−30.6 (3)
N2—C1—C2—C7	−171.7 (3)	Cu1—N2—C8—N1	144.60 (19)
N2—C1—C2—C3	11.3 (4)	C1—N2—C8—C9	147.5 (2)
Cu1—O1—C3—C4	149.6 (2)	Cu1—N2—C8—C9	−37.2 (3)
Cu1—O1—C3—C2	−32.7 (3)	N1—C8—C9—C10	0.8 (4)
C7—C2—C3—O1	−177.1 (2)	N2—C8—C9—C10	−177.2 (2)
C1—C2—C3—O1	−0.2 (4)	C8—C9—C10—C11	−0.1 (4)
C7—C2—C3—C4	0.7 (4)	C9—C10—C11—C12	0.0 (4)
C1—C2—C3—C4	177.6 (2)	C8—N1—C12—C11	1.5 (4)
O1—C3—C4—C5	177.8 (2)	C10—C11—C12—N1	−0.8 (5)
C2—C3—C4—C5	−0.1 (4)		

Symmetry codes: (i) −*x*+1, −*y*+1, −*z*+1.

### Hydrogen-bond geometry (Å, °)

**Table:**

*D*—H···*A*	*D*—H	H···*A*	*D*···*A*	*D*—H···*A*
C1—H1···N1	0.93	2.29	2.684 (3)	105
